# Successful rotational atherectomy of left main stem with double kiss crush stenting using double guiding catheter: a case report

**DOI:** 10.1093/ehjcr/ytab481

**Published:** 2021-12-04

**Authors:** Ghaith M Maqableh, Mohammed Osheiba, Anthony Mechery, Sohail Q Khan

**Affiliations:** 1 Department of Interventional Cardiology, Queen Elizabeth Hospital, University Hospitals Birmingham NHS Foundation Trust, Mindelsohn Way, Birmingham B15 2TH, UK; 2 Cardiology Department, Faculty of Medicine, Al Balqa Applied University, Amman, Jordan; 3 Cardiology Department, Faculty of Medicine, Tanta University, Tanta, Egypt; 4 Institute of Cardiovascular Sciences, University of Birmingham, Birmingham B15 2TT, UK

**Keywords:** Rotational atherectomy (RA), Double guiding catheter (Ping-Pong) technique, Coronary artery disease, Complex percutaneous coronary intervention (PCI), Double kiss crush stenting, Intravascular ultrasound (IVUS), Case report

## Abstract

**Background:**

Coronary artery bypass grafting is the preferred revascularization procedure for patients with multivessel disease, and those with complex left main disease, as it is associated with a survival advantage in this group of patients. Sometimes however surgical management is not the treatment of choice due to many factors including ongoing chest pain, haemodynamic instability, or patient preference. In these situations, percutaneous coronary intervention (PCI) offers an alternative revascularization strategy. In this case study, we present a successful PCI with rotational atherectomy (RA) for distal left main stem (LMS), left anterior descending (LAD), and circumflex artery (CX) using a double guide catheter technique in a patient with severe calcific disease.

**Case summary:**

A 63-year-old female was diagnosed with a non-ST-elevation myocardial infarction. Coronary angiography showed significant distal LMS disease with a severe proximal/ostial calcified lesion of the LAD and a possible thrombotic lesion at the ostium of the CX. She had ongoing haemodynamic instability with chest pain however could not be offered immediate surgical revascularization. We therefore elected to proceed to complex bifurcation LMS coronary intervention using RA under intravascular ultrasound (IVUS) guidance achieving an excellent final result with TIMI III flow.

**Discussion:**

This case demonstrates that RA using the double catheter technique (also known as Ping-Pong) can be safely performed with minimal complication rates and with very favourable angiographic and IVUS results. The clinical outcome was excellent with early discharge.

Learning points

- Rotational atherectomy (RA) using double catheter technique can facilitate interventional procedures in very complex heavily calcified lesions and can be safely accomplished with a very low complication rate.
- Double kiss crush stenting is a feasible two stent technique regardless of the bifurcation angle.
- Intracoronary imaging helps to plan complex bifurcation strategy.
- The ultra-low profile and braided nature of the Caravel microcatheter with its hydrophilic coating ensures that it is suitable to withstand high-speed RA.

## Introduction

The significant advances in percutaneous coronary intervention (PCI) over the past decade make it the most commonly used treatment modality for obstructive coronary artery disease. However, severe coronary calcification and multivessel disease remain a challenge.^1^

Surgical treatment is usually an attractive option for patients with multivessel disease, those with complex left main stem (LMS) disease and diabetic patients. Long-term data showed survival benefits for patients undergoing coronary artery bypass grafting (CABG) over PCI. Sometimes however surgical management is not always the treatment of choice due to many factors including ongoing chest pain, haemodynamic instability, or patient preference. In these situations, PCI offers an alternative revascularization strategy.^2^

## Timeline

**Table:** 

1st day	Admitted from the emergency department with 2-week history of anginal chest pain. Twelve-lead electrocardiogram (ECG): resting ST depression in V5–V6. High-sensitivity troponin I was 42 pg/mL (normal < 15 pg/mL)
2nd day	Transthoracic echocardiogram: Normal biventricular size and systolic function. Ejection fraction 68%. No regional wall motion abnormalities No significant valvular abnormality
3rd day	Diagnostic coronary angiogram was undertaken, followed by percutaneous coronary intervention to left main stem, left anterior descending and circumflex artery with the resolution of symptoms and ECG changes.
5th day	Discharged from hospital. Remains asymptomatic.

## Case presentation

A 63-year-old female was admitted via the emergency department following a 2-week episode of anginal chest pain. Her only significant past medical history is hypertension. She is a non-smoker, retired and independently mobile. Her physical examination was unremarkable. Her 12-lead electrocardiogram (ECG) showed sinus rhythm with T-wave inversion in the antero-lateral leads. She had a significant elevation in her high-sensitive troponin I (42 pg/mL; normal < 15 pg/mL) and her echocardiogram revealed normal biventricular size and systolic function. In view of her history, she was brought forward for coronary angiography. The procedure was performed initially from the right radial approach after administration of 5000 units of heparin.

**Figure 1:**
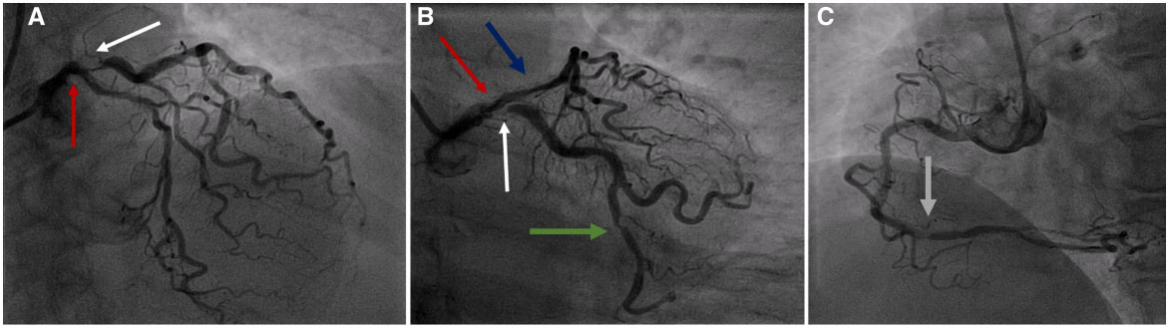
Coronary angiography showing significant distal left main stem disease (red arrow) with severe ostio-proximal calcific disease of the left anterior descending (blue arrow), and severe ostial calcific disease of the circumflex artery (white arrow) with further moderate distal disease of AV circumflex artery (green arrow) in cranial (*A*) and caudal (*B*) views. The right coronary artery showed distal moderate calcific disease (grey arrow) in left anterior oblique view (*C*).

Coronary angiography (*Figure 1*) showed heavily calcified vessels. The right coronary artery was dominant with moderate stenosis distally. The LMS was atheromatous but there was no pressure damping on catheter engagement. There was a lesion in the proximal circumflex which appeared thrombotic. There was a large Obtuse marginal artery (OM) branch and further disease in the Atrioventricular (AV) circumflex which had (90%) stenosis. Left anterior descending (LAD) was calcified with significant stenosis in the proximal and mid vessel.

Following the angiogram, the patient developed chest pain with ST-segment depression on her ECG. A detailed discussion had been undertaken with the patient prior to the procedure and her preference was to undergo PCI. However, given the complex anatomy, we felt it was necessary to discuss this case with cardiac surgery. Unfortunately, due to lack of intensive therapy unit (ITU) capacity and urgent surgical slots, they were unable to offer her immediate CABG and given the patients preference for PCI, we therefore elected to proceed to coronary intervention. Her calculated SYNTAX II scores for PCI and CABG were 29% and 20.7%, respectively. Her predicted 4-year mortality for PCI was 6.4%, and 3.2% for CABG.

She was loaded with 180 mg Ticagrelor and we administered further heparin during the procedure with a plan to target an Activated Clotting Time (ACT) of 300.

**Figure 2:**
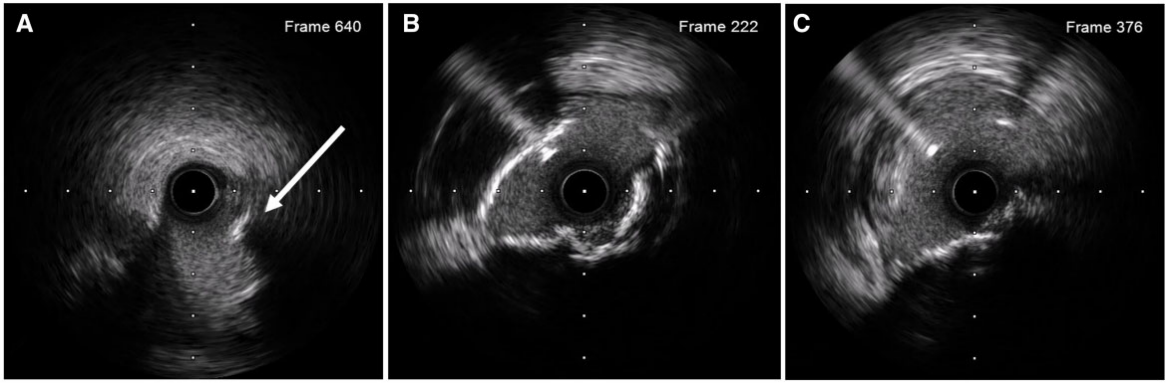
Intravascular ultrasound of the circumflex confirmed a calcified nodule (white arrow) with a mild thrombotic lesion with a minimal luminal area of 1.2 mm^2^ (*A*). Ostial disease of the left anterior descending with 270° arc of calcification and minimal luminal area of 4.8 mm^2^ (*B*) and the minimal luminal area of the left main stem was 5.9 mm^2^ (*C*).

Intravascular ultrasound (IVUS) of the circumflex in fact confirmed a calcified nodule with minimal thrombus and a minimal luminal area of 1.2 mm^2^. There was also disease at the ostium of the LAD with 270° arc of calcification and minimal luminal area of 4.8 mm^2^ the minimal luminal area of LMS 5.9 mm^2^ (*Figure 2*, *Video 1*, *2*, supplementary Video 1).

Given the IVUS finding, we felt that high pressure balloon dilatation of the circumflex would increase the risk of perforation. Given the small amount of thrombus, we accepted a small risk of distal embolization. We decided to perform rotablation to the LAD initially; however, we did not want to remove the circumflex wire, so a second voda left 3.5 guide catheter was introduced through a 7-Fr femoral sheath with a plan for double catheter rotablation.

The LAD was wired through the femoral guide and we switched out to a Rota wire over a Caravel microcatheter (ASAHI). The circumflex artery (CX) was wired through the radial guide and we protected the circumflex wire with the same microcatheter. We proceeded to successfully burr the LAD with a 1.75 rotaburr at 175 000 rpm through the femoral guide. Following this, the circumflex was burred with the same 1.75 rotaburr at 175 000 rpm, this time through the radial guide having protected the LAD with the microcatheter.

There was a very good angiographic result following rotablation and we proceeded to double kiss crush (DK-Crush) stenting of the LMS with a 3.5 × 19 drug-eluting stent (DES) to the circumflex and a 3.5 × 33 DES from LMS into LAD. An excellent result was achieved with TIMI III flow in both vessels (*Video 3*).

**Figure 3:**
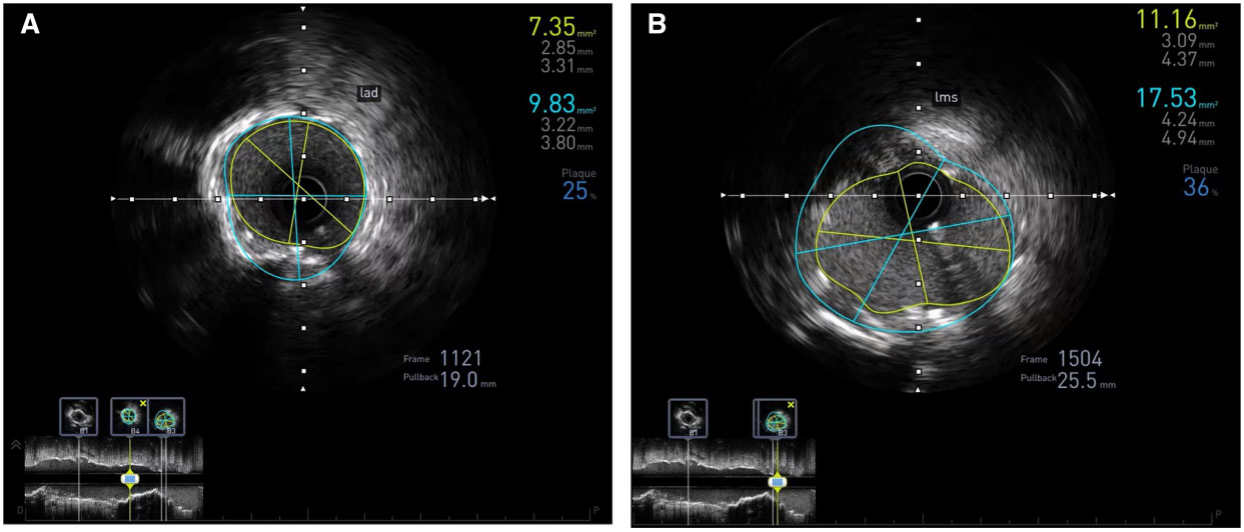
Post-procedure intravascular ultrasound of the left anterior descending (*A*) and left main stem (*B*) showing well-apposed stent struts with a minimal luminal area of 7.35 mm^2^ and 11.16 mm^2^, respectively.

Intravascular ultrasound of the LAD confirmed well-apposed stent struts with a minimal luminal area of 7.35 mm^2^, LMS area was above 11 mm^2^ (*Figure 3*).

The femoral artery was sealed with an angio-seal and radial by a TR band.

The patient was discharged home 2 days later on lifelong aspirin 75 mg o.d. and ticagrelor 90 mg b.i.d. for 1 year. In addition, standard acute coronary syndrome treatment was prescribed (atorvastatin 80 mg o.d., bisoprolol 5 mg o.d., perindopril 2 mg o.d., and sublingual glyceryl trinitrate as required).

At clinic review, she remained asymptomatic and has been medically managed for her residual coronary disease. She remains under follow-up.

## Discussion

In actual practice, there are a lot of factors that usually prevent patients with complex lesions or multivessel disease from undergoing open heart surgery. This case study demonstrates the use of rotational atherectomy (RA) using double guide catheter technique as a primary strategy for treating heavily calcified LAD and CX lesions in order to facilitate optimal stent delivery and expansion.

Our patient had multiple tight calcified lesions; such lesions are not well treated with high pressure balloon inflation due to risk of barotrauma and vessel rupture. Stent delivery through calcified lesions may also be difficult and stent expansion may be suboptimal due to high resistance of the calcified plaques. Rotational atherectomy is particularly useful in this situation as it allows plaque modification to facilitate successfully stent delivery.^3^

Intravascular lithotripsy (IVL) is also an option for the treatment of heavily calcified lesions; however, IVL in the LMS would involve obstruction of the LMS for short periods of time which may lead to haemodynamic instability.

**Figure 4:**
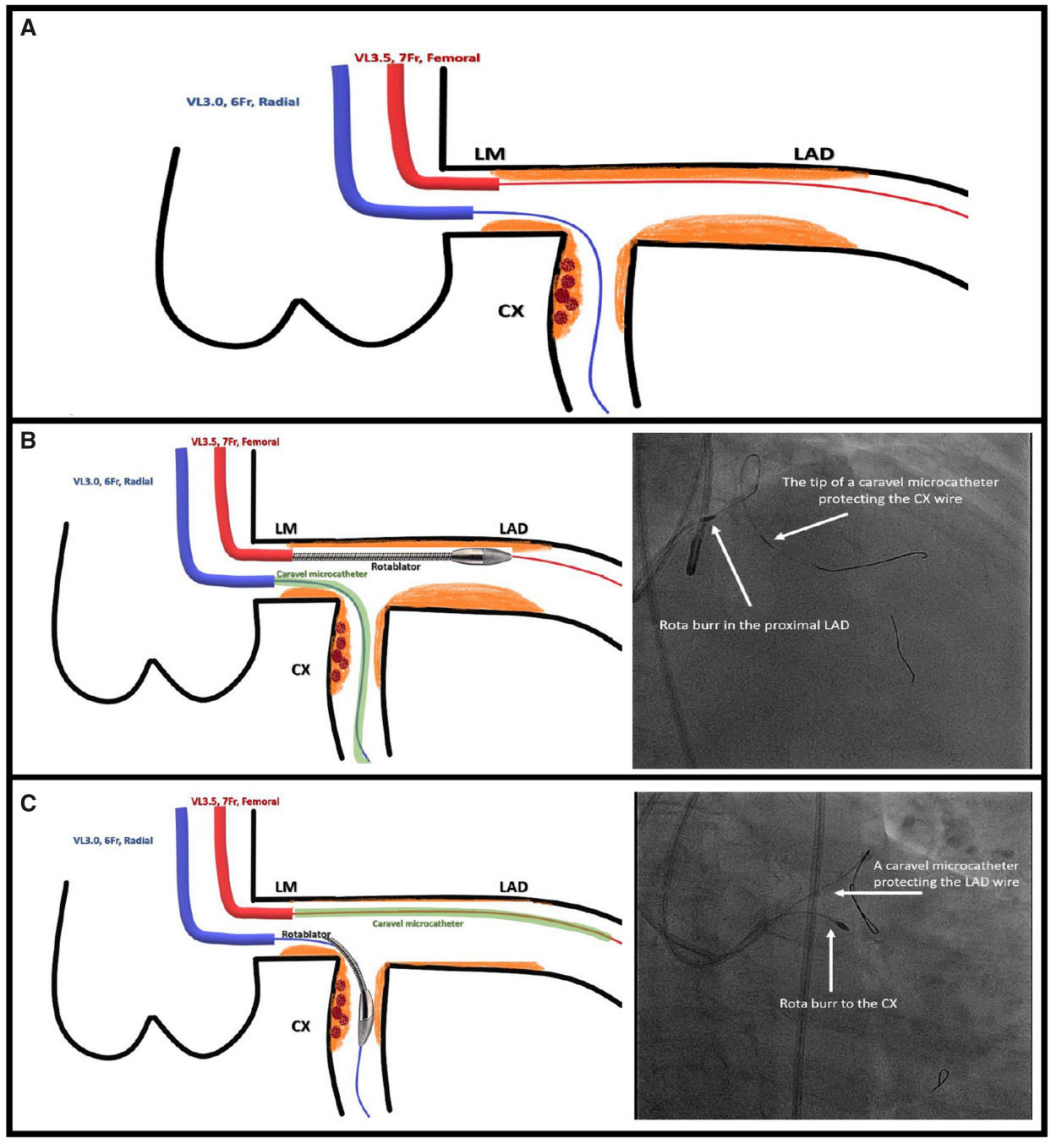
The double guide catheter technique. A 6 Fr, voda left 3.0 radial guide catheter (blue) was used to wire the circumflex artery, and a 7 Fr voda left 3.5 femoral guide catheter (red) was used to wire the left anterior descending (*A*). The circumflex artery wire was protected by a Caravel microcatheter, whilst rotational atherectomy was undertaken on the left anterior descending (*B*). The left anterior descending wire was protected with the same Caravel microcatheter, whilst rotational atherectomy was undertaken to the (*C*).

One of the most important steps in RA is removing any additional side branch wire before commencing rotablation to avoid guidewire fracture or entrapment.^4^ In our case, we did not wish to remove our circumflex wire as we were concerned that after RA to the LAD, we could lose access to this important branch. To prevent this scenario occurring, a second guide catheter was introduced from the femoral approach with a plan for double catheter rotablation.^5^^,^^6^ Additionally, to avoid CX wire fracture during the rotablation, the CX wire was protected with a Caravel microcatheter (ASAHI); its low profile and hydrophilic polymer coating provide excellent flexibility and guidewire protection (*Figure 4*).

Double guide catheter technique has been shown to be useful in a variety of other clinical scenarios including sealing of coronary perforations or rupture and treatment of chronic total occlusions.

After RA of LMS, LAD, and CX, we proceeded to DK-Crush stenting, which is the preferred bifurcation strategy in our centre. This technique has shown superiority over other bifurcation stenting techniques in terms of restenosis and stent thrombosis, especially for the LMS.^7^

Intravascular ultrasound was used before DK-Crush stenting to help clarify the proximal circumflex lesion composition and aid choice of calcium modification tools. It also assisted in determining appropriate burr size for rotablation, choosing appropriate stent length and diameter. Further stent optimization was achieved by the use of post-procedural IVUS to achieve better stent expansion and improved stent apposition.^8^

## Conclusion

We have demonstrated that double access to facilitate RA is feasible. This is a technique for the treatment of very complex heavily calcified distal left main lesions which allows protection of the side branch. Additionally, we opted for IVUS-guided DK-Crush stenting which is associated with a significant improvement in clinical outcomes in patients with complex coronary bifurcation lesions compared with the provisional stenting approach.

## Lead author biography

**Figure:**
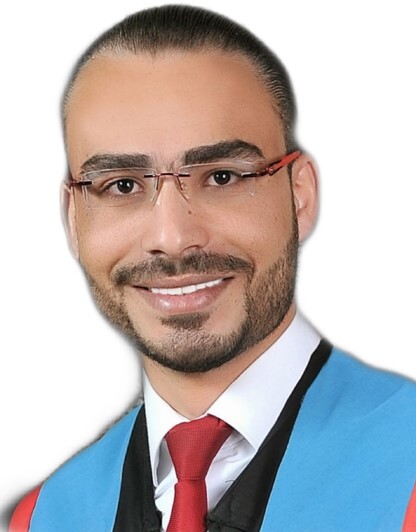


Dr Ghaith M. Maqableh qualified as an Internal Medicine Specialist from Jordan Medical Council in 2019. He graduated with a higher specialty certificate in Internal Medicine from Jordan University of Science and Technology—King Abdullah University Hospital. He is currently undertaking a 3-year fellowship in general and interventional cardiology which commenced in 2021 at the Queen Elizabeth Hospital (University Hospitals Birmingham NHS Foundation Trust) under the supervision of Dr Sohail Q. Khan.

## Supplementary material

Supplementary material is available at *European Heart Journal - Case Reports* online.

**Slide sets:** A fully edited slide set detailing this case and suitable for local presentation is available online as Supplementary data.

**Consent:** The authors confirm that written consent for the submission and publication of this case report including images and associated text has been obtained from the patient in line with COPE guidance.

**Conflict of interest:** None declared.

**Funding:** None declared.

## Supplementary Material

ytab481_Supplementary_Data
