# Supplementary material for: Successful rotational atherectomy of left main stem with double kiss crush stenting using double guiding catheter: a case report
Source: Eur Heart J Case Rep. 2021 Dec 4;5(12):ytab481. doi: 10.1093/ehjcr/ytab481 (PMC8759481; doi:10.1093/ehjcr/ytab481)
Supplement: ytab481_Supplementary_Data [file ytab481_supplementary_data.zip › Slide Set November 2021.pptx]

## Slide 1
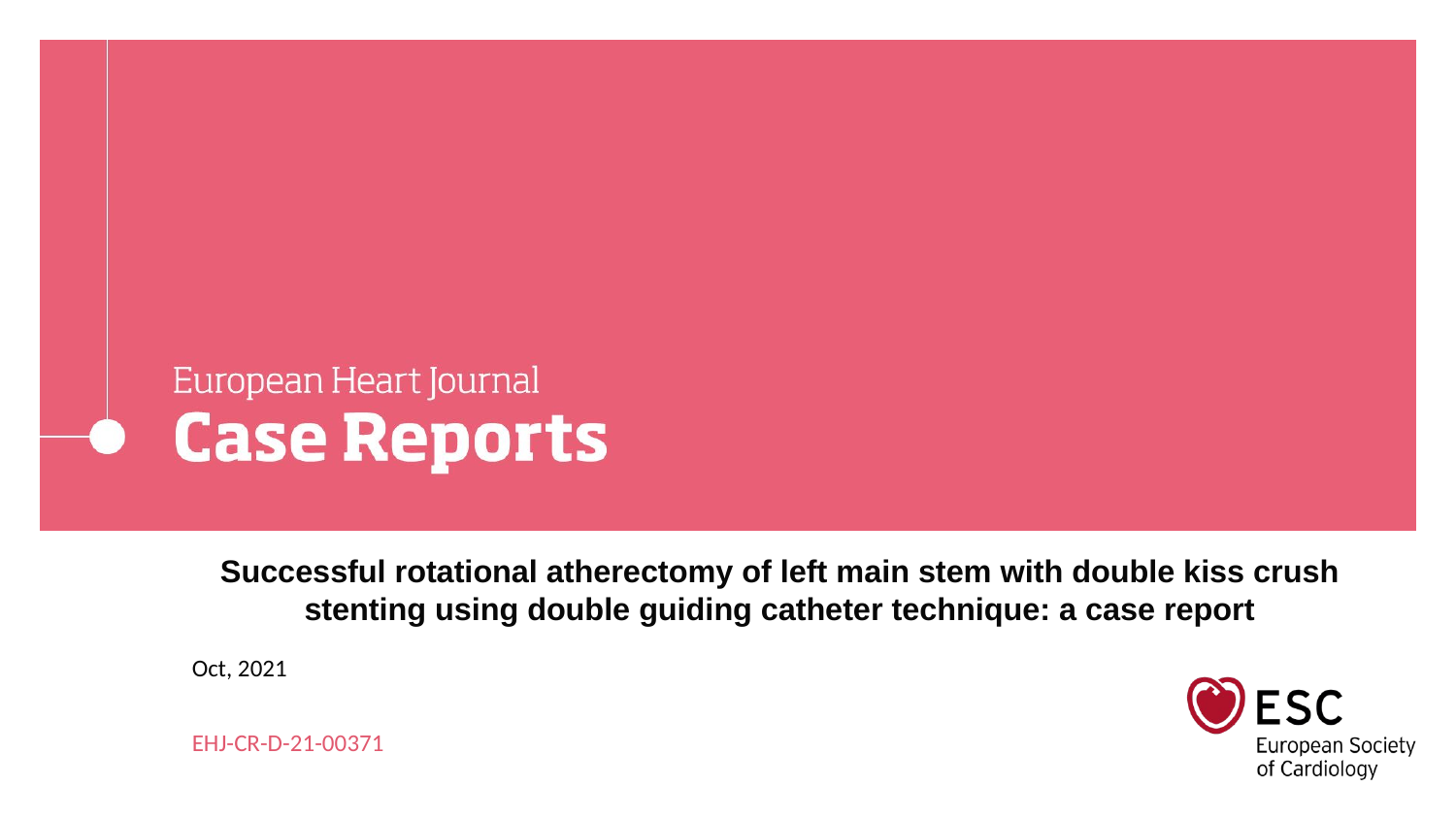

# Successful rotational atherectomy of left main stem with double kiss crush stenting using double guiding catheter technique: a case report
Oct, 2021
EHJ-CR-D-21-00371

## Slide 2
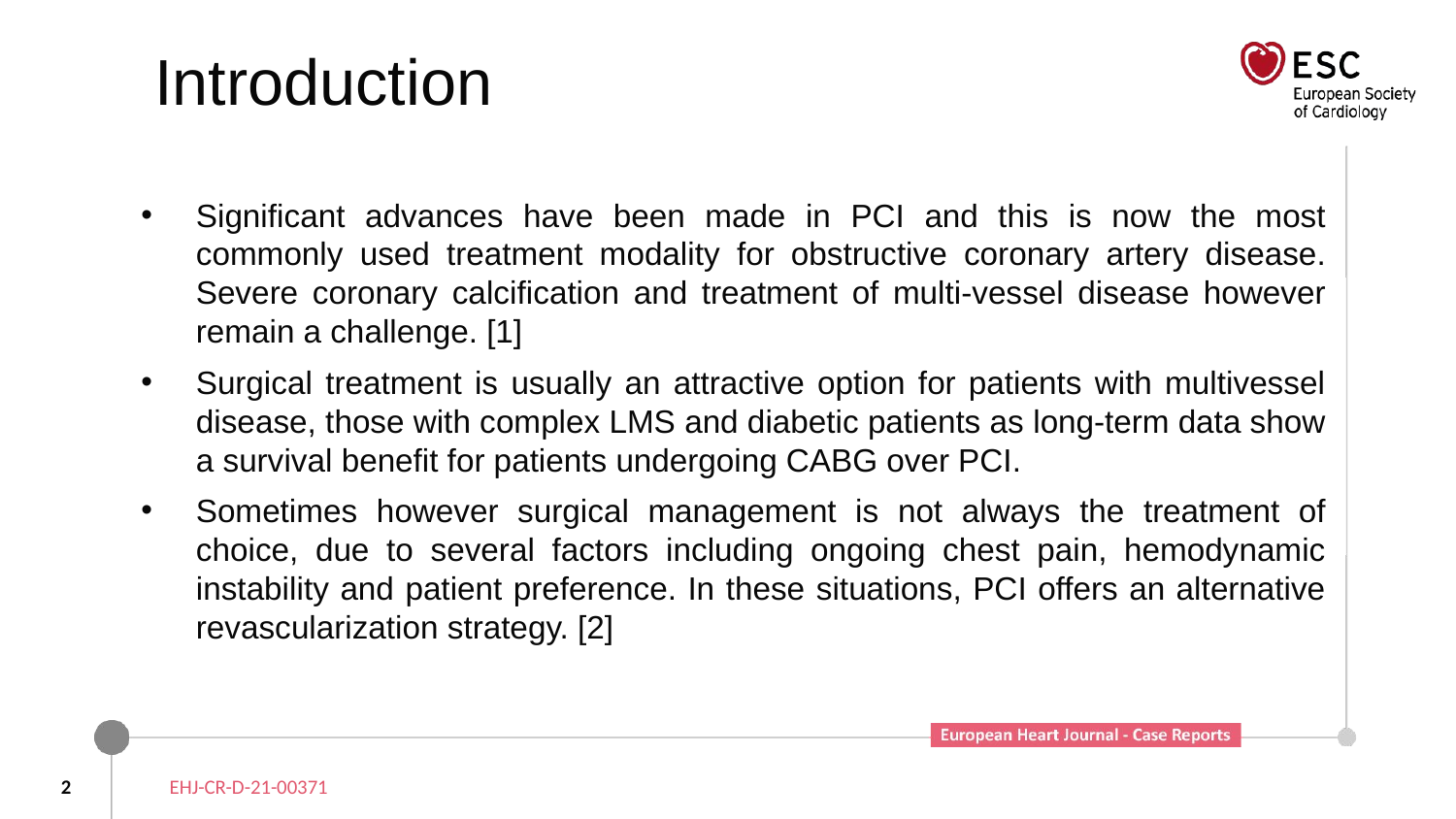

# Introduction
Significant advances have been made in PCI and this is now the most commonly used treatment modality for obstructive coronary artery disease. Severe coronary calcification and treatment of multi-vessel disease however remain a challenge. [1]
Surgical treatment is usually an attractive option for patients with multivessel disease, those with complex LMS and diabetic patients as long-term data show a survival benefit for patients undergoing CABG over PCI.
Sometimes however surgical management is not always the treatment of choice, due to several factors including ongoing chest pain, hemodynamic instability and patient preference. In these situations, PCI offers an alternative revascularization strategy. [2]
2
EHJ-CR-D-21-00371

## Slide 3
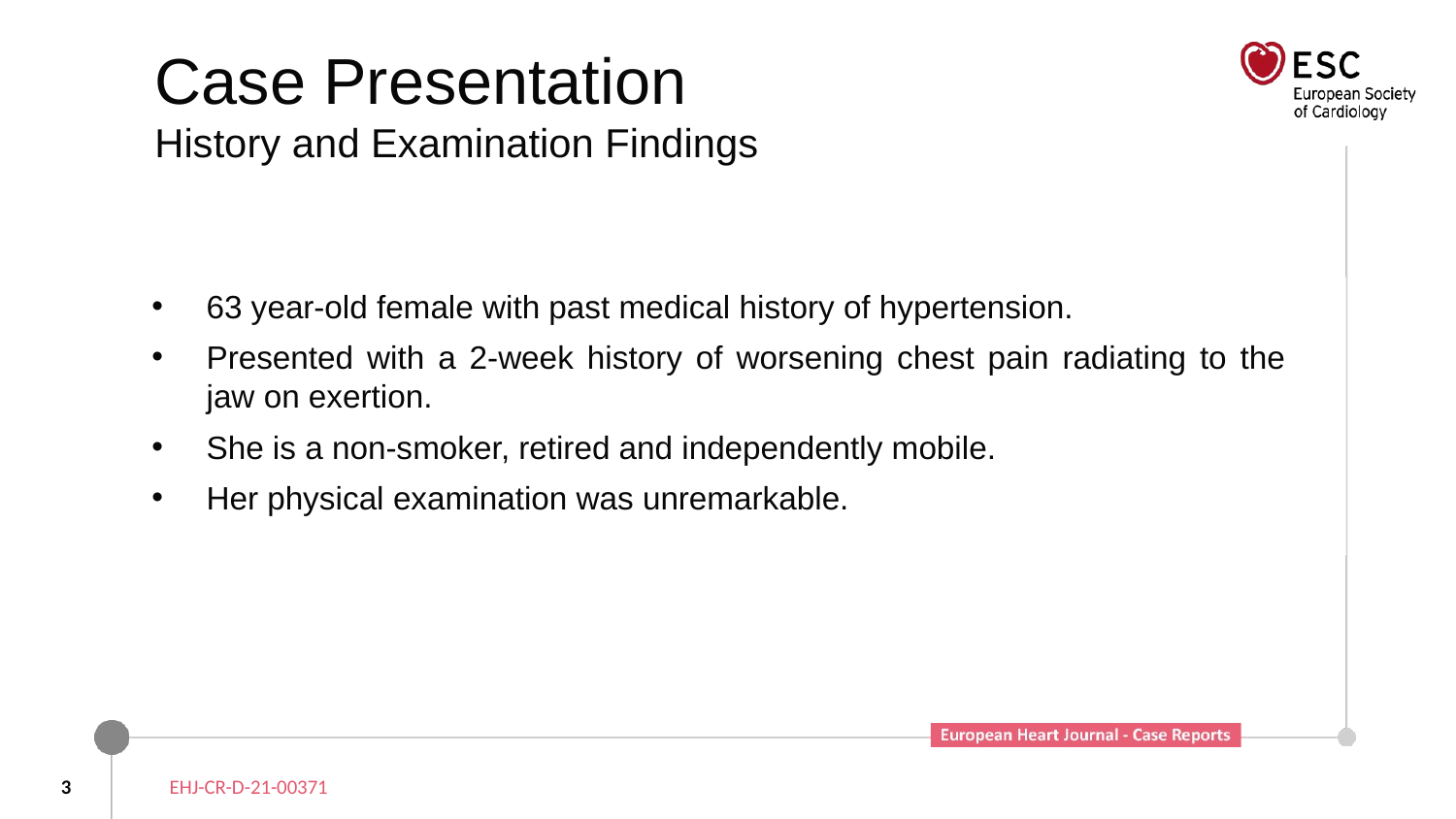

# Case Presentation History and Examination Findings
63 year-old female with past medical history of hypertension.
Presented with a 2-week history of worsening chest pain radiating to the jaw on exertion.
She is a non-smoker, retired and independently mobile.
Her physical examination was unremarkable.
3
EHJ-CR-D-21-00371

## Slide 4
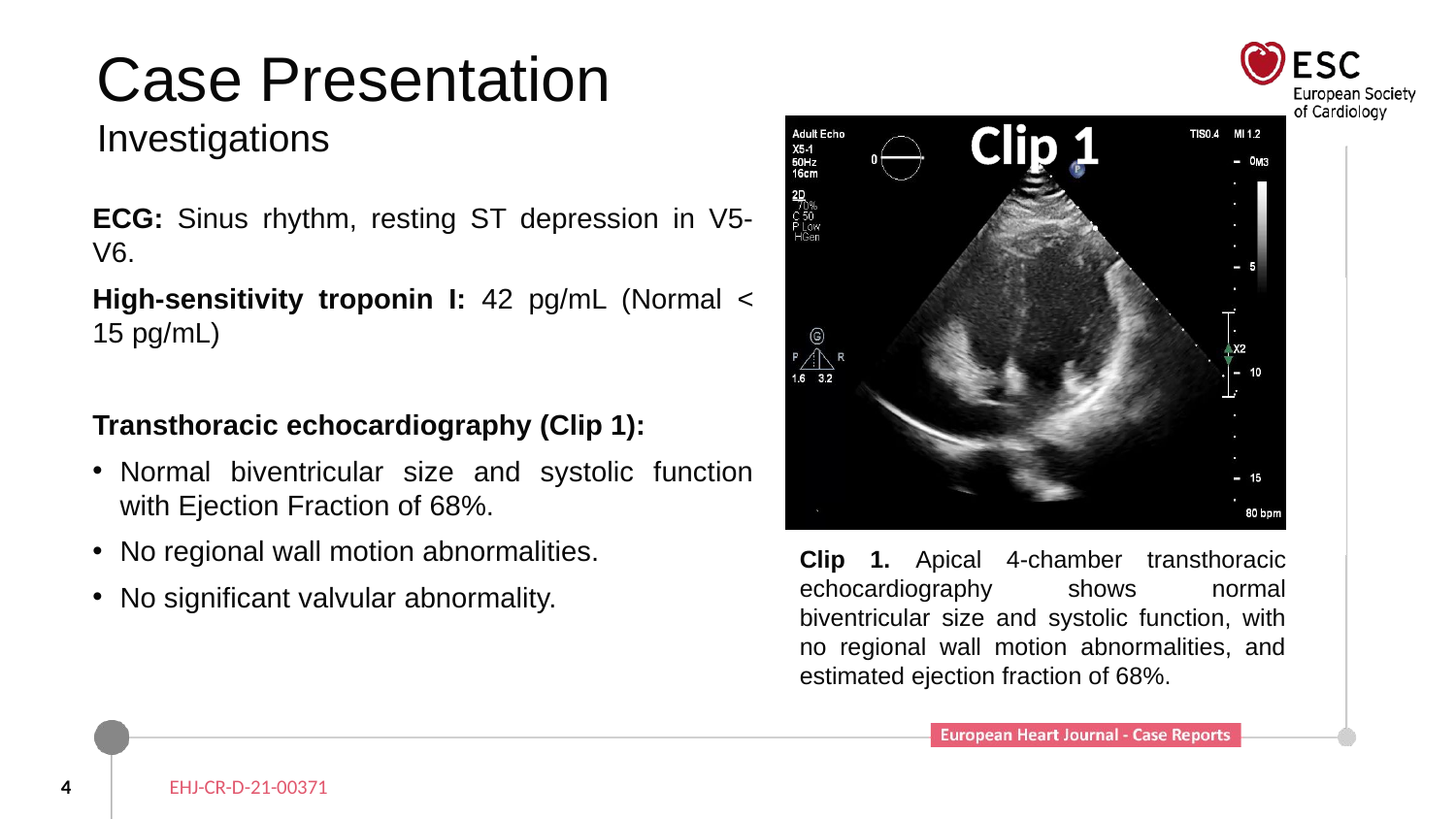

# Case Presentation Investigations
Clip 1
ECG: Sinus rhythm, resting ST depression in V5-V6.
High-sensitivity troponin I: 42 pg/mL (Normal < 15 pg/mL)
Transthoracic echocardiography (Clip 1):
Normal biventricular size and systolic function with Ejection Fraction of 68%.
No regional wall motion abnormalities.
No significant valvular abnormality.
Clip 1. Apical 4-chamber transthoracic echocardiography shows normal biventricular size and systolic function, with no regional wall motion abnormalities, and estimated ejection fraction of 68%.
4
EHJ-CR-D-21-00371

## Slide 5
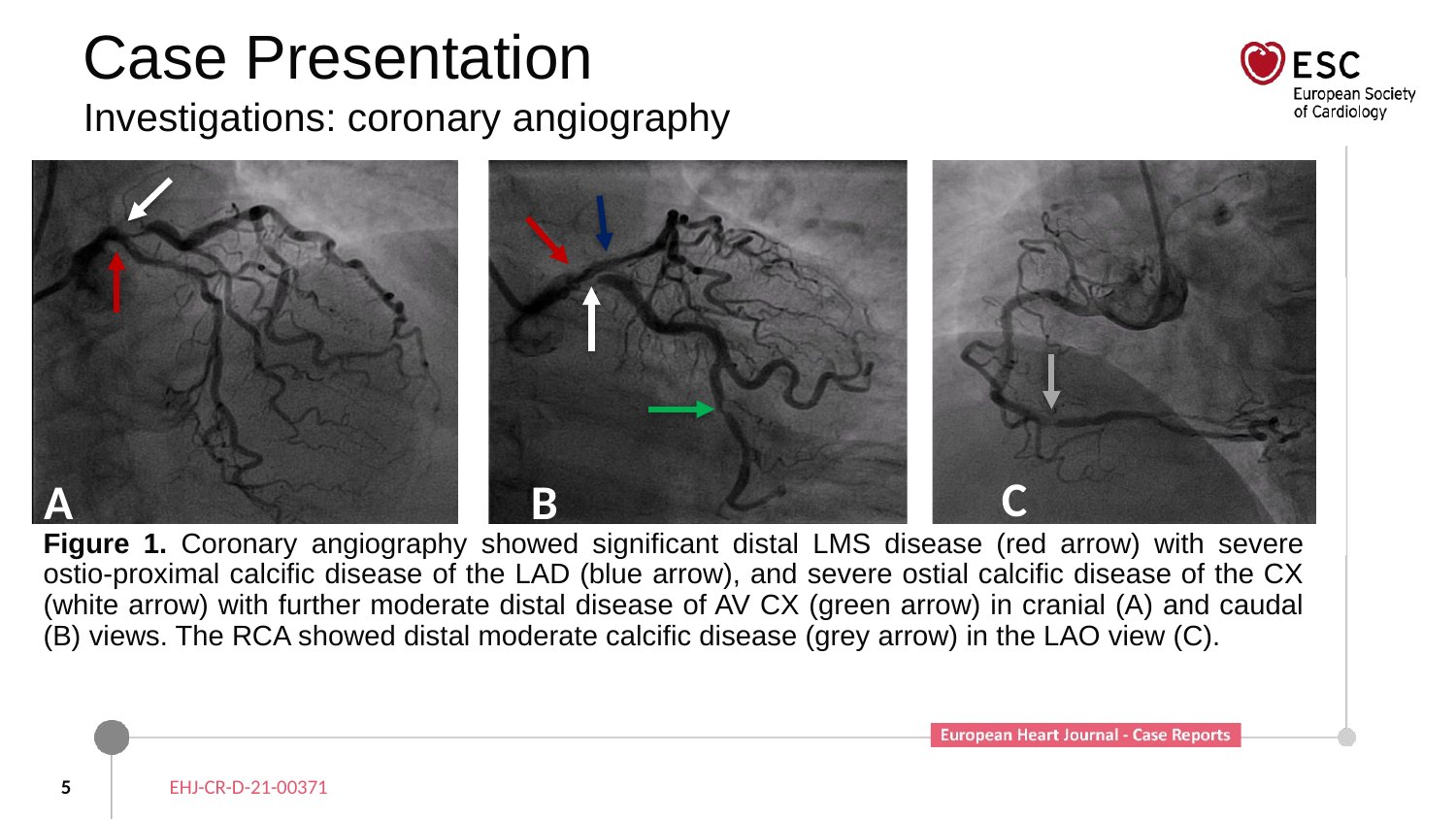

Case Presentation
Investigations: coronary angiography
C
A
B
Figure 1. Coronary angiography showed significant distal LMS disease (red arrow) with severe ostio-proximal calcific disease of the LAD (blue arrow), and severe ostial calcific disease of the CX (white arrow) with further moderate distal disease of AV CX (green arrow) in cranial (A) and caudal (B) views. The RCA showed distal moderate calcific disease (grey arrow) in the LAO view (C).
5
EHJ-CR-D-21-00371

## Slide 6
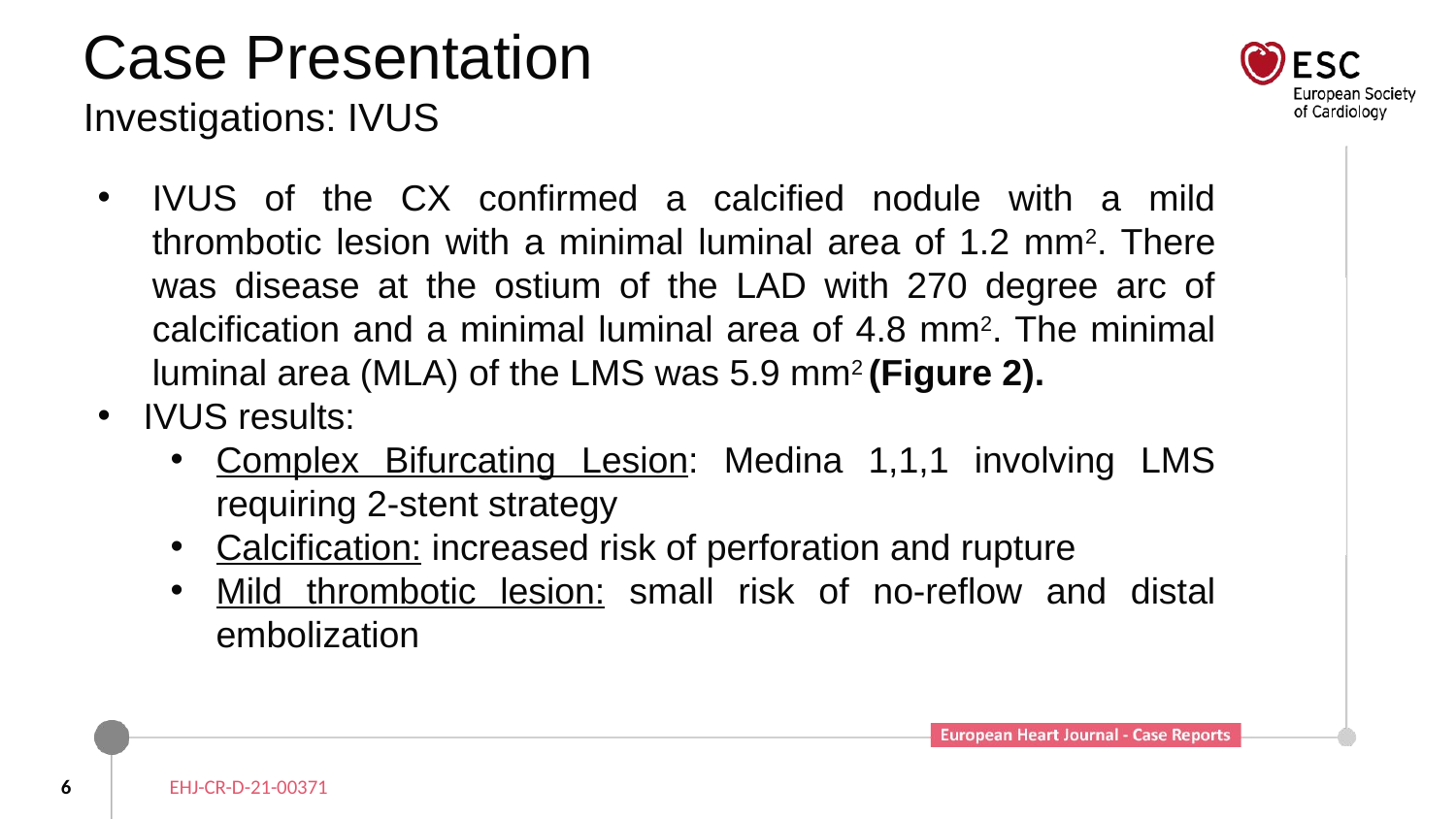

Case Presentation
Investigations: IVUS
IVUS of the CX confirmed a calcified nodule with a mild thrombotic lesion with a minimal luminal area of 1.2 mm2. There was disease at the ostium of the LAD with 270 degree arc of calcification and a minimal luminal area of 4.8 mm2. The minimal luminal area (MLA) of the LMS was 5.9 mm2 (Figure 2).
IVUS results:
Complex Bifurcating Lesion: Medina 1,1,1 involving LMS requiring 2-stent strategy
Calcification: increased risk of perforation and rupture
Mild thrombotic lesion: small risk of no-reflow and distal embolization
B
6
EHJ-CR-D-21-00371

## Slide 7
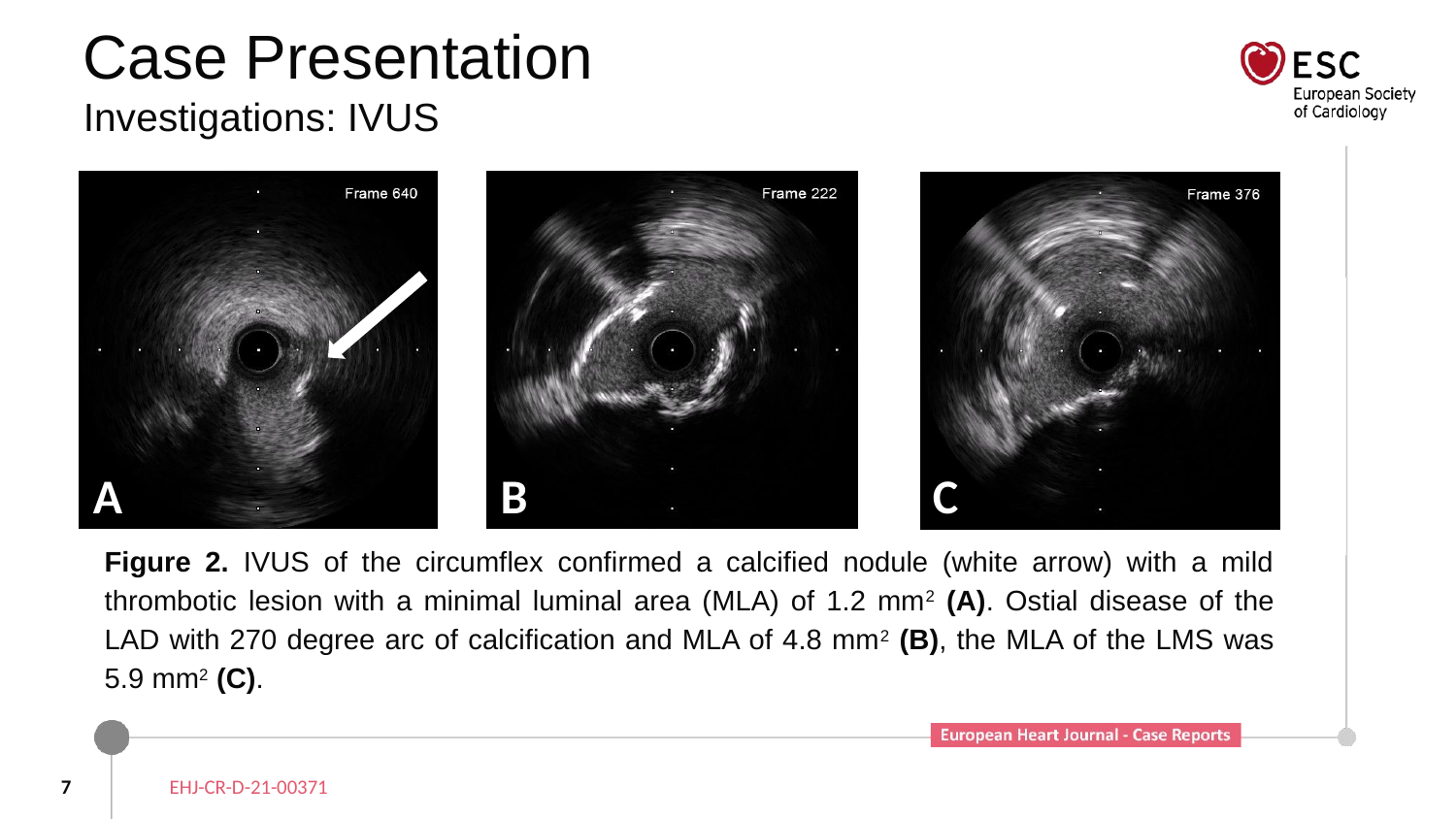

Case Presentation
Investigations: IVUS
C
A
B
Figure 2. IVUS of the circumflex confirmed a calcified nodule (white arrow) with a mild thrombotic lesion with a minimal luminal area (MLA) of 1.2 mm2 (A). Ostial disease of the LAD with 270 degree arc of calcification and MLA of 4.8 mm2 (B), the MLA of the LMS was 5.9 mm2 (C).
7
EHJ-CR-D-21-00371

## Slide 8
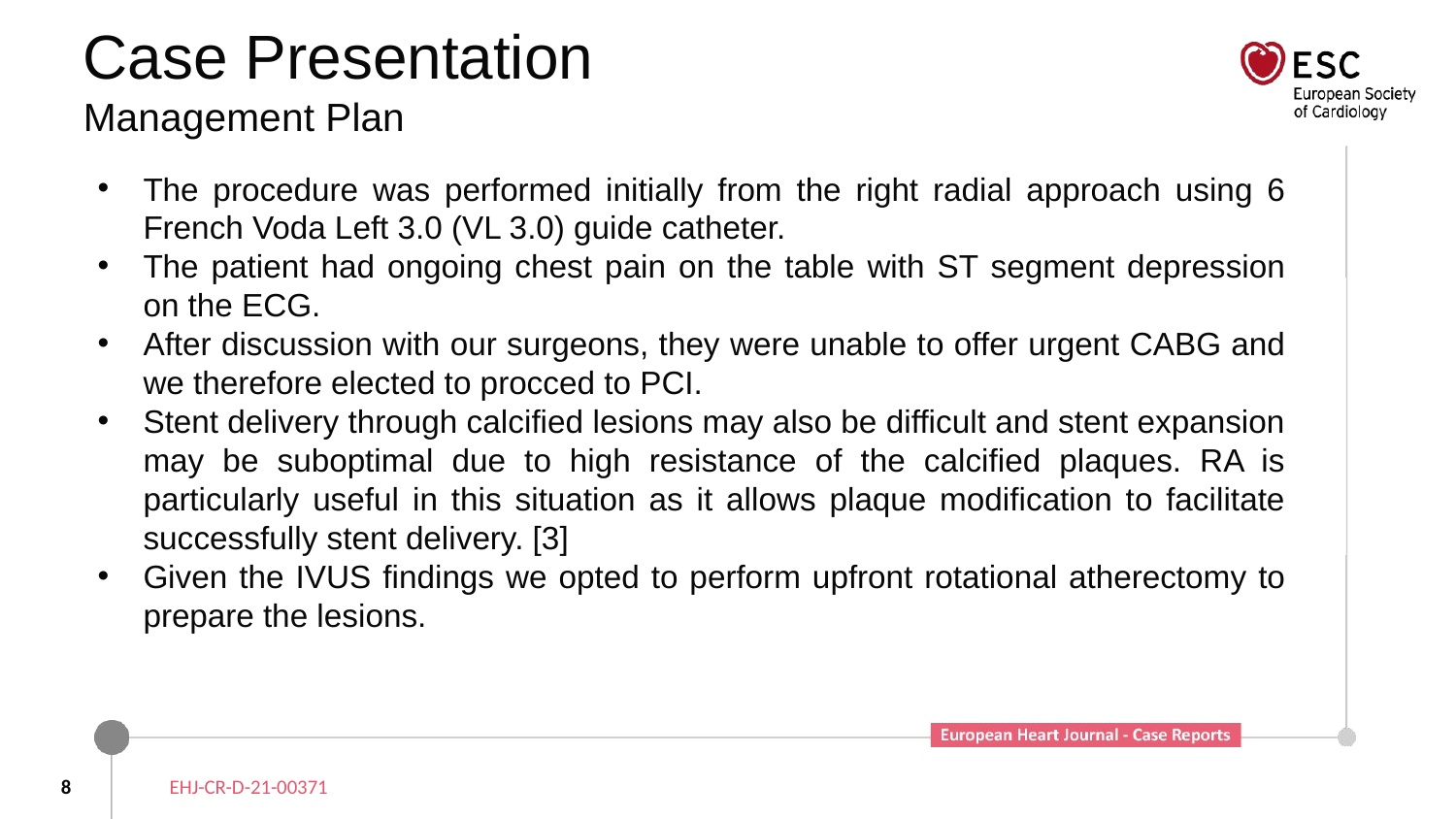

Case Presentation
Management Plan
The procedure was performed initially from the right radial approach using 6 French Voda Left 3.0 (VL 3.0) guide catheter.
The patient had ongoing chest pain on the table with ST segment depression on the ECG.
After discussion with our surgeons, they were unable to offer urgent CABG and we therefore elected to procced to PCI.
Stent delivery through calcified lesions may also be difficult and stent expansion may be suboptimal due to high resistance of the calcified plaques. RA is particularly useful in this situation as it allows plaque modification to facilitate successfully stent delivery. [3]
Given the IVUS findings we opted to perform upfront rotational atherectomy to prepare the lesions.
8
EHJ-CR-D-21-00371

## Slide 9
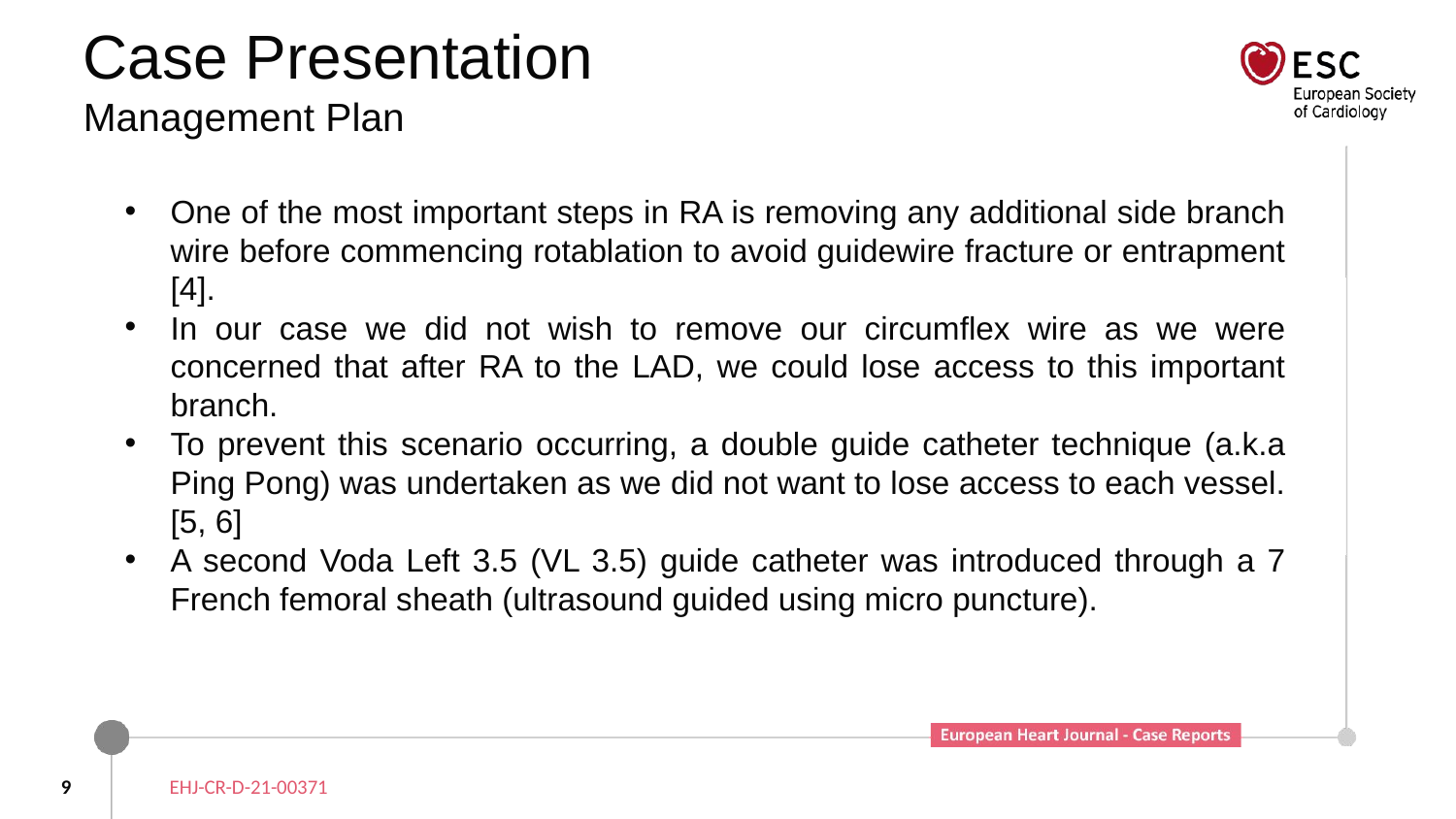

Case Presentation
Management Plan
One of the most important steps in RA is removing any additional side branch wire before commencing rotablation to avoid guidewire fracture or entrapment [4].
In our case we did not wish to remove our circumflex wire as we were concerned that after RA to the LAD, we could lose access to this important branch.
To prevent this scenario occurring, a double guide catheter technique (a.k.a Ping Pong) was undertaken as we did not want to lose access to each vessel. [5, 6]
A second Voda Left 3.5 (VL 3.5) guide catheter was introduced through a 7 French femoral sheath (ultrasound guided using micro puncture).
9
EHJ-CR-D-21-00371

## Slide 10
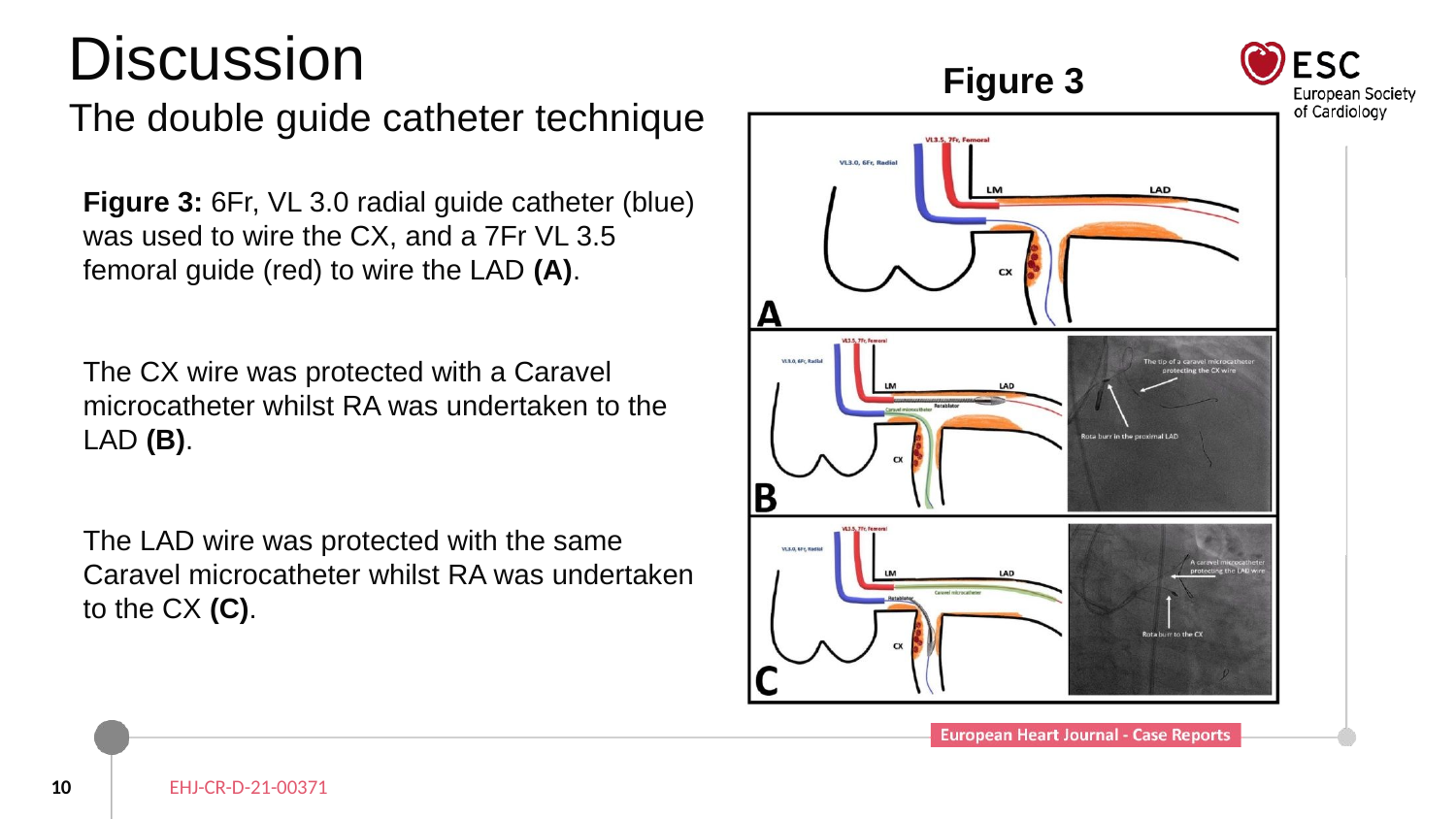

Discussion
The double guide catheter technique
Figure 3
Figure 3: 6Fr, VL 3.0 radial guide catheter (blue) was used to wire the CX, and a 7Fr VL 3.5 femoral guide (red) to wire the LAD (A).
The CX wire was protected with a Caravel microcatheter whilst RA was undertaken to the LAD (B).
The LAD wire was protected with the same Caravel microcatheter whilst RA was undertaken to the CX (C).
10
EHJ-CR-D-21-00371

## Slide 11
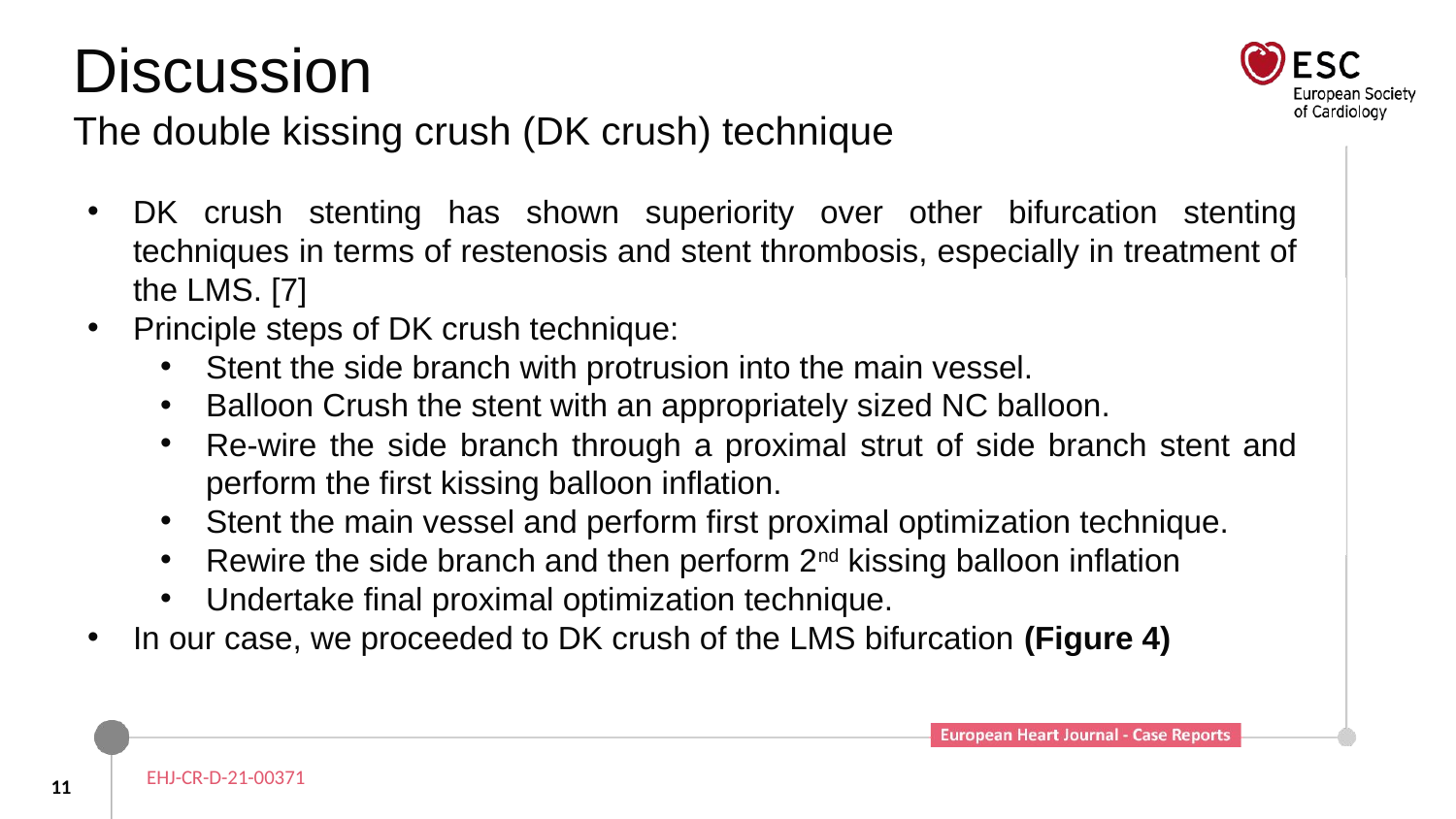

Discussion
The double kissing crush (DK crush) technique
DK crush stenting has shown superiority over other bifurcation stenting techniques in terms of restenosis and stent thrombosis, especially in treatment of the LMS. [7]
Principle steps of DK crush technique:
Stent the side branch with protrusion into the main vessel.
Balloon Crush the stent with an appropriately sized NC balloon.
Re-wire the side branch through a proximal strut of side branch stent and perform the first kissing balloon inflation.
Stent the main vessel and perform first proximal optimization technique.
Rewire the side branch and then perform 2nd kissing balloon inflation
Undertake final proximal optimization technique.
In our case, we proceeded to DK crush of the LMS bifurcation (Figure 4)
EHJ-CR-D-21-00371
11

## Slide 12
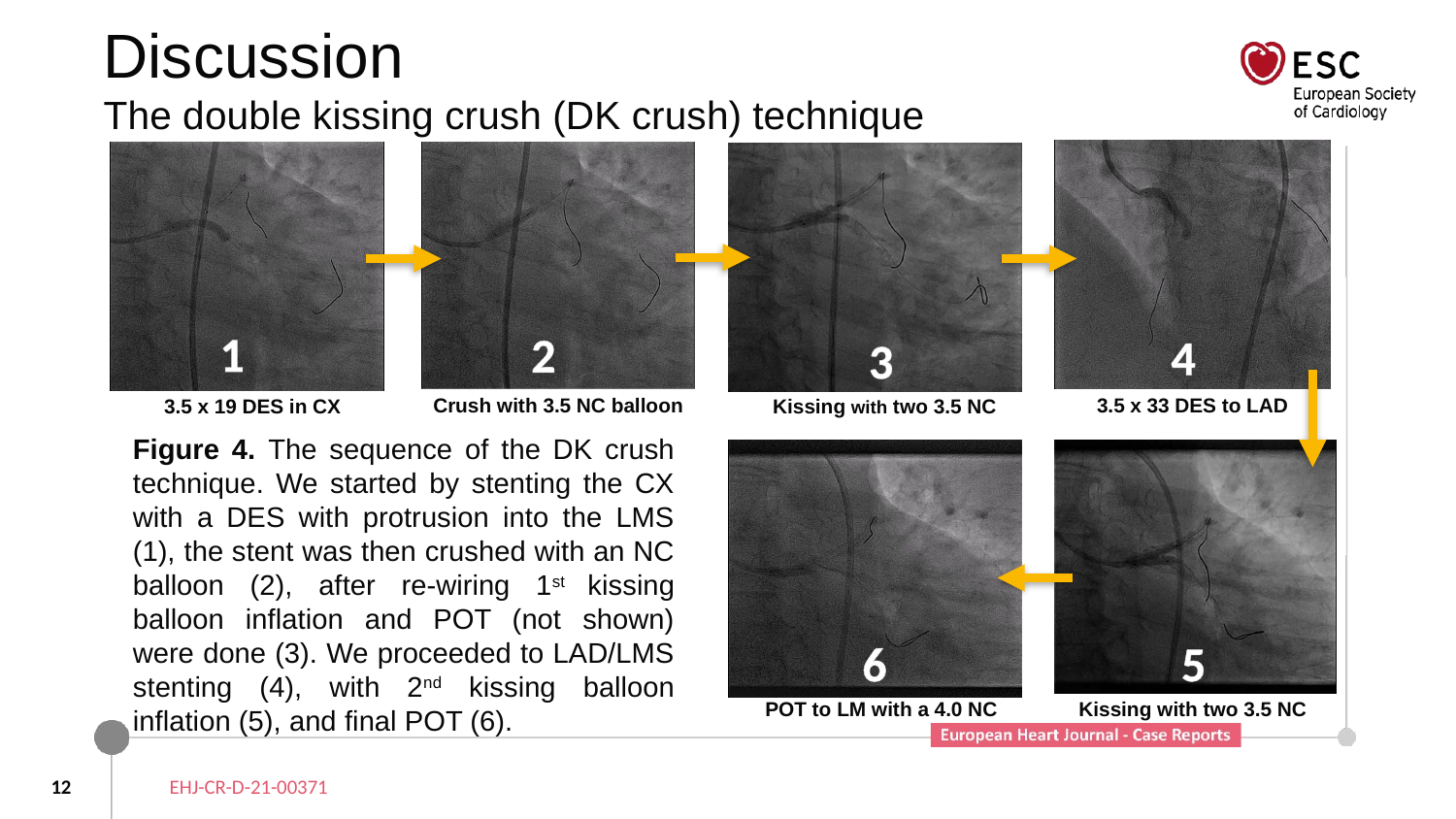

Discussion
The double kissing crush (DK crush) technique
1
2
4
3
3.5 x 33 DES to LAD
Crush with 3.5 NC balloon
3.5 x 19 DES in CX
Kissing with two 3.5 NC
Figure 4. The sequence of the DK crush technique. We started by stenting the CX with a DES with protrusion into the LMS (1), the stent was then crushed with an NC balloon (2), after re-wiring 1st kissing balloon inflation and POT (not shown) were done (3). We proceeded to LAD/LMS stenting (4), with 2nd kissing balloon inflation (5), and final POT (6).
5
6
Kissing with two 3.5 NC
POT to LM with a 4.0 NC
12
EHJ-CR-D-21-00371

## Slide 13
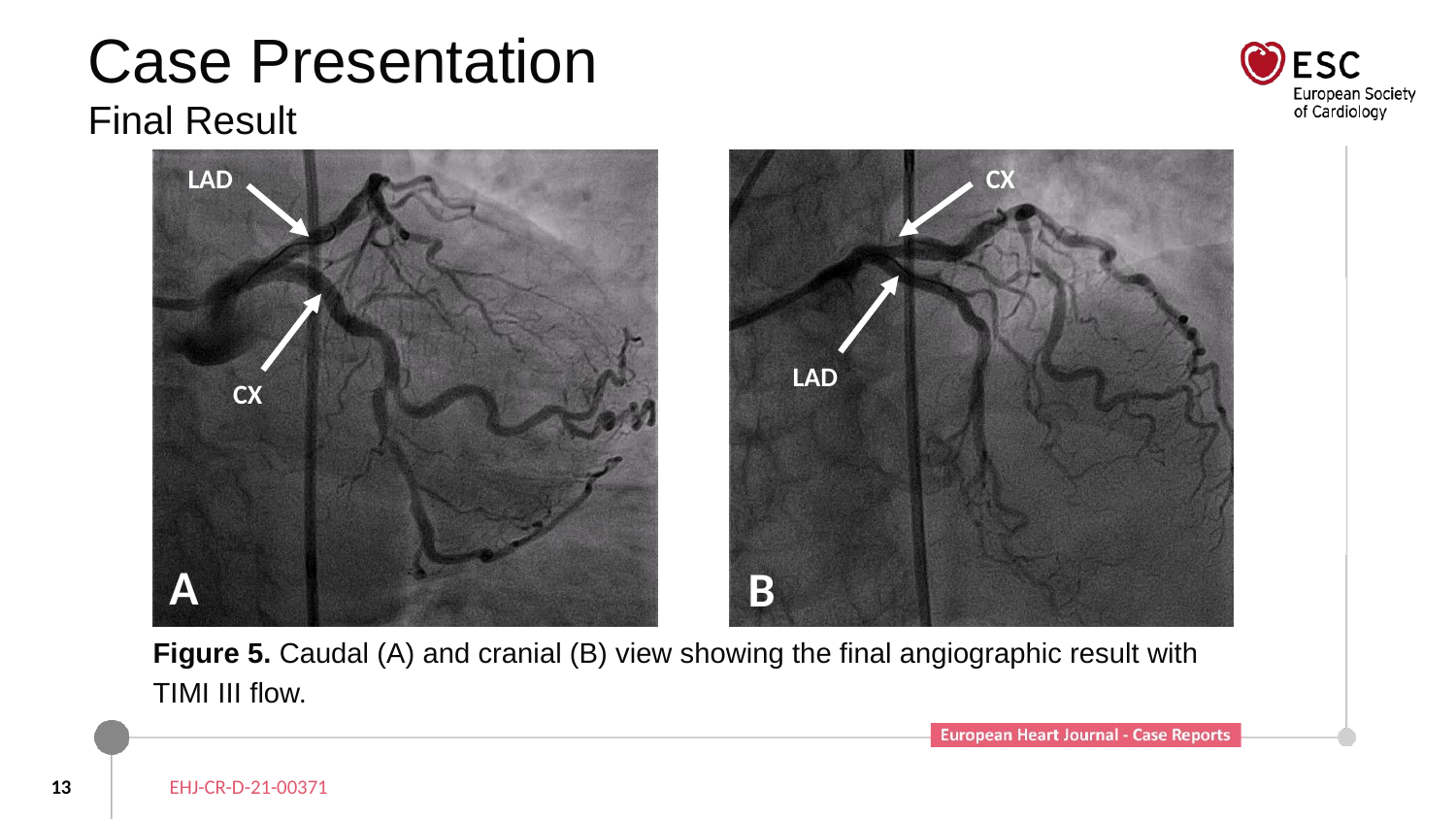

Case Presentation
Final Result
LAD
CX
LAD
CX
A
B
Figure 5. Caudal (A) and cranial (B) view showing the final angiographic result with TIMI III flow.
13
EHJ-CR-D-21-00371

## Slide 14
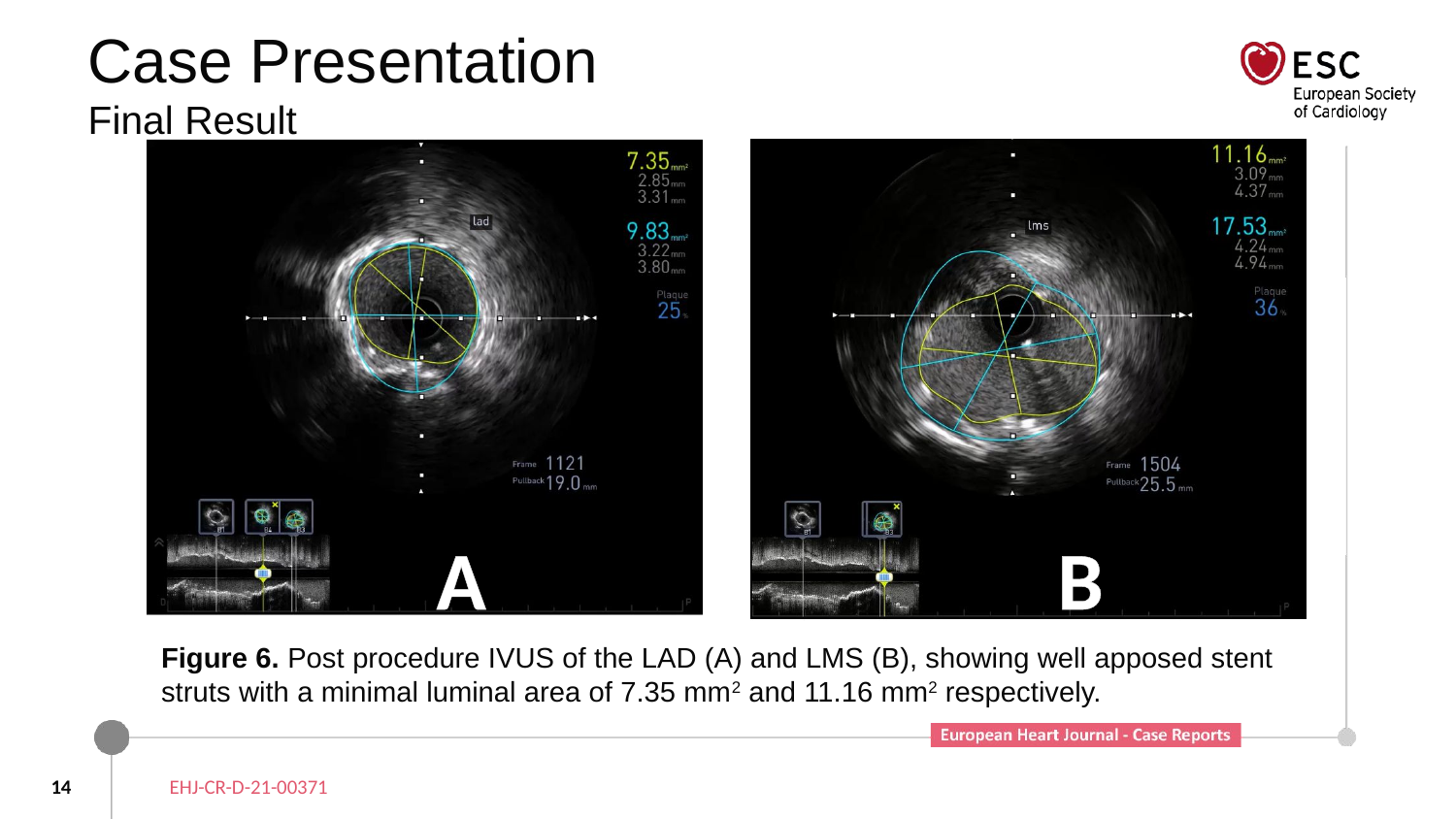

Case Presentation
Final Result
A
B
Figure 6. Post procedure IVUS of the LAD (A) and LMS (B), showing well apposed stent struts with a minimal luminal area of 7.35 mm2 and 11.16 mm2 respectively.
14
EHJ-CR-D-21-00371

## Slide 15
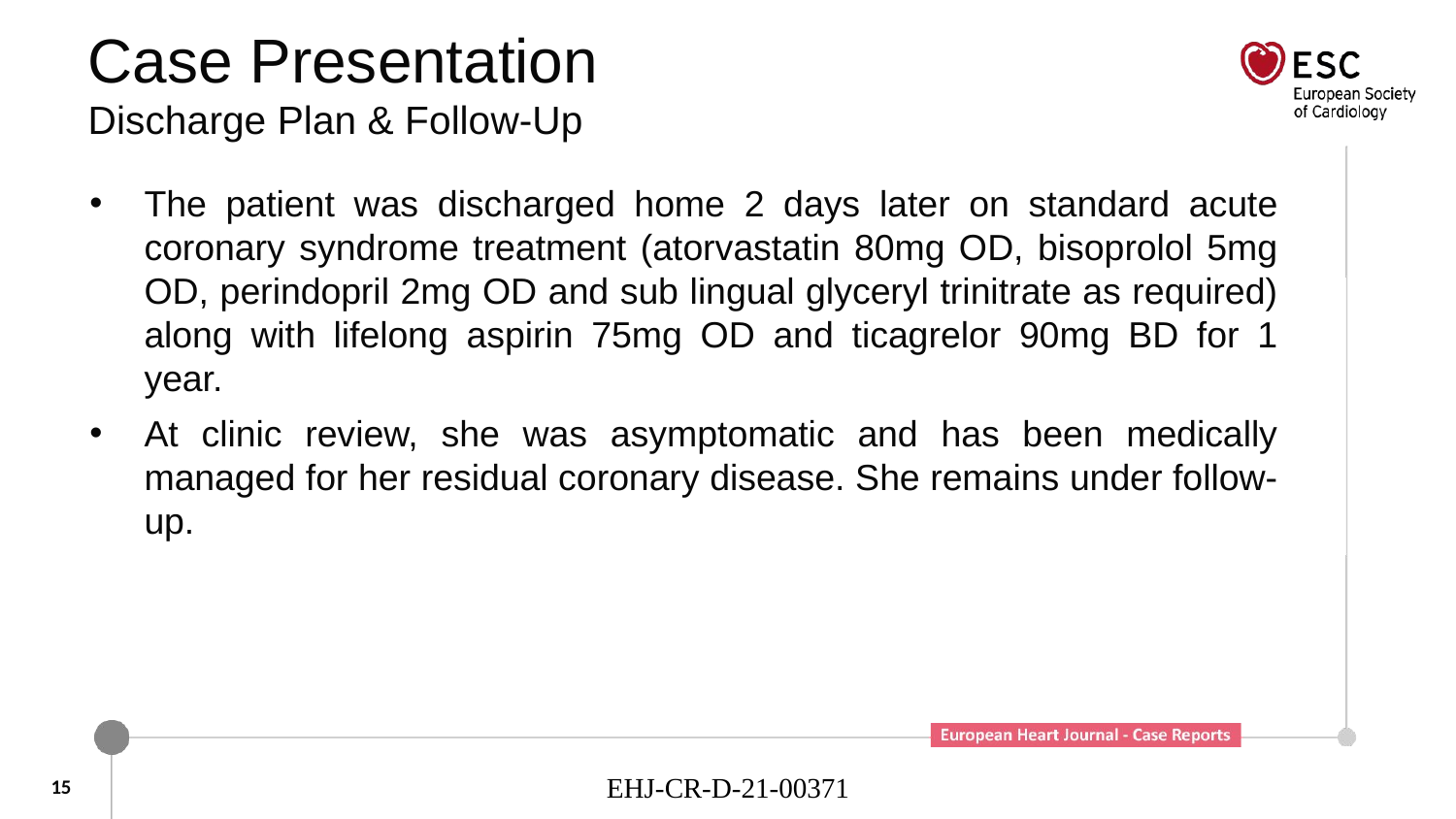

Case Presentation
Discharge Plan & Follow-Up
The patient was discharged home 2 days later on standard acute coronary syndrome treatment (atorvastatin 80mg OD, bisoprolol 5mg OD, perindopril 2mg OD and sub lingual glyceryl trinitrate as required) along with lifelong aspirin 75mg OD and ticagrelor 90mg BD for 1 year.
At clinic review, she was asymptomatic and has been medically managed for her residual coronary disease. She remains under follow-up.
15
EHJ-CR-D-21-00371

## Slide 16
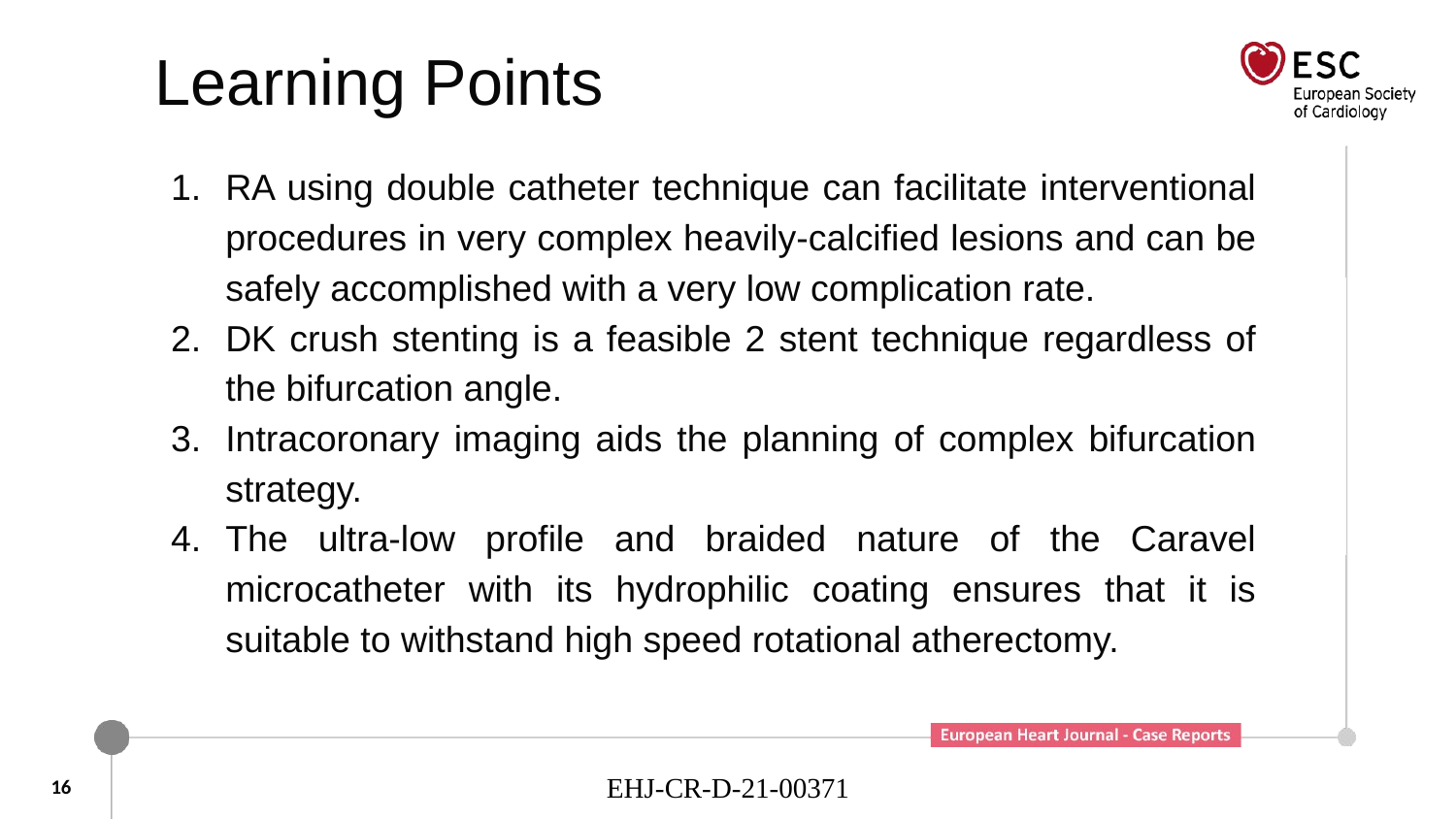

# Learning Points
RA using double catheter technique can facilitate interventional procedures in very complex heavily-calcified lesions and can be safely accomplished with a very low complication rate.
DK crush stenting is a feasible 2 stent technique regardless of the bifurcation angle.
Intracoronary imaging aids the planning of complex bifurcation strategy.
The ultra-low profile and braided nature of the Caravel microcatheter with its hydrophilic coating ensures that it is suitable to withstand high speed rotational atherectomy.
16
EHJ-CR-D-21-00371

## Slide 17
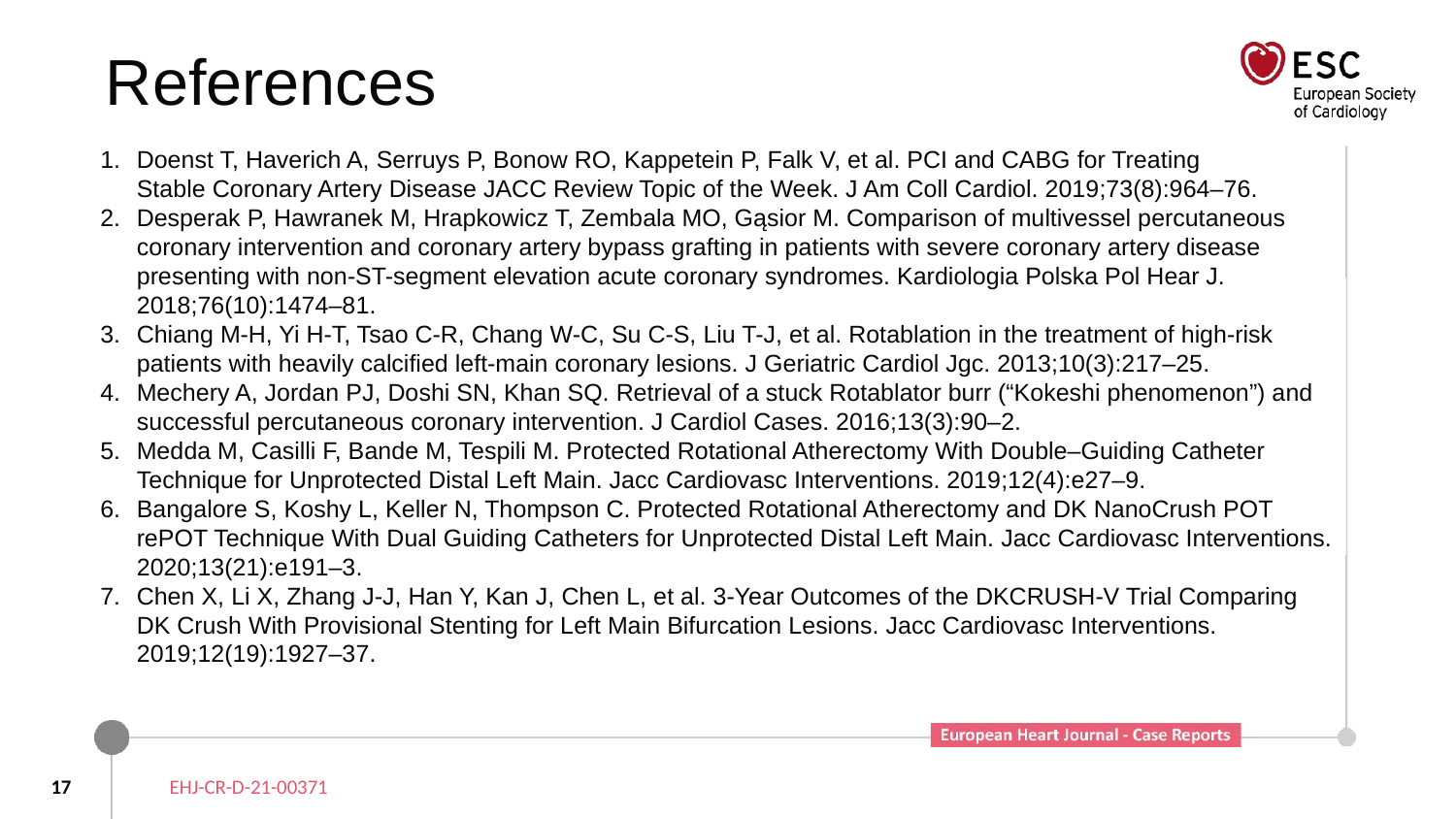

# References
Doenst T, Haverich A, Serruys P, Bonow RO, Kappetein P, Falk V, et al. PCI and CABG for Treating Stable Coronary Artery Disease JACC Review Topic of the Week. J Am Coll Cardiol. 2019;73(8):964–76.
Desperak P, Hawranek M, Hrapkowicz T, Zembala MO, Gąsior M. Comparison of multivessel percutaneous coronary intervention and coronary artery bypass grafting in patients with severe coronary artery disease presenting with non-ST-segment elevation acute coronary syndromes. Kardiologia Polska Pol Hear J. 2018;76(10):1474–81.
Chiang M-H, Yi H-T, Tsao C-R, Chang W-C, Su C-S, Liu T-J, et al. Rotablation in the treatment of high-risk patients with heavily calcified left-main coronary lesions. J Geriatric Cardiol Jgc. 2013;10(3):217–25.
Mechery A, Jordan PJ, Doshi SN, Khan SQ. Retrieval of a stuck Rotablator burr (“Kokeshi phenomenon”) and successful percutaneous coronary intervention. J Cardiol Cases. 2016;13(3):90–2.
Medda M, Casilli F, Bande M, Tespili M. Protected Rotational Atherectomy With Double–Guiding Catheter Technique for Unprotected Distal Left Main. Jacc Cardiovasc Interventions. 2019;12(4):e27–9.
Bangalore S, Koshy L, Keller N, Thompson C. Protected Rotational Atherectomy and DK NanoCrush POT rePOT Technique With Dual Guiding Catheters for Unprotected Distal Left Main. Jacc Cardiovasc Interventions. 2020;13(21):e191–3.
Chen X, Li X, Zhang J-J, Han Y, Kan J, Chen L, et al. 3-Year Outcomes of the DKCRUSH-V Trial Comparing DK Crush With Provisional Stenting for Left Main Bifurcation Lesions. Jacc Cardiovasc Interventions. 2019;12(19):1927–37.
17
EHJ-CR-D-21-00371
